# Critical Comparison of the Quality and Content of Integrated Vascular Surgery, Thoracic Surgery, and Interventional Radiology Residency Training Program Websites: Qualitative Study

**DOI:** 10.2196/35074

**Published:** 2022-06-29

**Authors:** Katherine Jensen, Qi Yan, Mark G Davies

**Affiliations:** 1 Division of Vascular and Endovascular Surgery University of Texas Health at San Antonio San Antonio, TX United States

**Keywords:** training, recruitment, website, content, quality, vascular surgery, thoracic surgery, interventional radiology, radiology, surgery, web-based, web resource, surgeon, comparison, residency, integrated program

## Abstract

**Background:**

With the move to virtual interviewing, residency websites are an important recruitment resource, introducing applicants to programs across the country and allowing for comparison. Recruitment is highly competitive from a common potential pool between vascular surgery, thoracic surgery, and interventional radiology with the ratio of applicants to positions being highest in interventional radiology, followed by thoracic surgery and lastly vascular surgery, as reported by the National Resident Matching Program.

**Objective:**

The aim of this study is to evaluate the accessibility and availability of online content for those integrated residency programs.

**Methods:**

A list of accredited vascular surgery, thoracic surgery, and interventional radiology residencies was obtained from the Accreditation Council for Graduate Medical Education (ACGME) database. Program websites were evaluated by trained independent reviewers (n=2) for content items pertaining to program recruitment and education (scored absent or present). Statistical analysis was performed in R software.

**Results:**

Of ACGME-accredited programs, 56 of 61 (92%) vascular surgery, 27 of 27 (100%) thoracic surgery, and 74 of 85 (87%) interventional radiology programs had functional websites (*P*=.12). Vascular surgery websites contained a median of 26 (IQR 20-32) content items, thoracic surgery websites contained a median of 27 (IQR 21-32) content items, and interventional radiology websites contained a median of 23 (IQR 18-27) content items. Two content items considered highly influential to applicant program decisions are procedural experience and faculty mentorship, which were reported at 32% (18/56) and 11% (6/56) for vascular surgery, 19% (5/27) and 11% (3/27) for thoracic surgery, and 50% (37/74) and 15% (11/74) for interventional radiology (*P*=.008 and *P*=.75), respectively. Key deficits were work hours, debt management, and curriculum for interventional radiology; resident profiles, sample contracts, and research interests in vascular surgery; and operative experiences and the program director’s contact and message for thoracic surgery. Interventional radiology deficits were work hours, and thoracic surgery deficits were procedural experience. Both interventional radiology and thoracic surgery websites lacked information on evaluation criteria and faculty mentorship.

**Conclusions:**

This study has uncovered key differences in the availability of online content for residencies recruiting from the same pool of applicants. Thoracic surgery has the most information, followed by vascular surgery, with interventional radiology reporting the least content. In the era of virtual interviewing from the same potential pool of applicants, programs should review and revise their web presence with the aim to increase the availability of online content to attract valuable candidates.

## Introduction

The role of vascular surgeons in the medical environment has changed considerably with the increasing use of endovascular approaches for treatment of vascular lesions [1]. By 2026, it is predicted that 75% to 95% of overall vascular lesions (aneurysms, stenosis, occlusive disease, traumatic vascular lesions, etc) will be treated endovascularly [1,2]. Vascular surgery, as always, will continue to compete in recruitment with cardiac surgery for procedural domain, but with the increasing use of endovascular approaches, it faces additional recruitment competition from interventional radiology [2]. Due to the overlap in patient populations, professional interests, skills, and treatments performed by vascular surgeons, thoracic surgeons, and interventional radiologists, these specialties appeal to a common potential applicant pool, and recruitment is highly competitive among these training programs.

Candidates for residency programs increasingly use the internet to research potential programs for application [3-6]. Online information has been analyzed for a range of residency and fellowship programs, including orthopedic surgery, plastic and reconstructive surgery, emergency medicine, cardiothoracic surgery, neurosurgery, otolaryngology, trauma surgery, surgical critical care, acute care surgery, microsurgery, interventional radiology, and vascular surgery [3-5,7-27]. Studies have individually analyzed the availability of online content for integrated vascular surgery [27], thoracic surgery [12], and interventional radiology [15,25] training program websites, but to our knowledge, no study has compared the accessibility and availability of online content across these training paradigms. Given the importance of online resources in recruiting prospective applicants and the current mandates to move to virtual interviewing, we sought to assess the current state of integrated vascular surgery, thoracic surgery, and interventional radiology training program websites. The purpose of this study is to evaluate the presence, accessibility, and comprehensiveness of integrated vascular surgery, thoracic surgery, and interventional radiology training program websites.

## Methods

### Study Design

A comprehensive list of accredited integrated vascular surgery, thoracic surgery, and interventional radiology residencies was obtained from the Accreditation Council for Graduate Medical Education (ACGME) database. Programs participating in the 2020 National Resident Matching Program (NRMP) were eligible for study inclusion. Following identification of all programs with websites, programs were accessed and evaluated by two independent reviewers (one medical student and one resident) for availability of recruitment and educational content items. The websites were viewed independently by each reviewer. The program search and review was performed in November 2019.

### Research Question

Are there key differences in the three specialty program websites for integrated residencies that could potentially impair recruitment efforts in the virtual environment?

### Accessibility of Websites

Accessibility of websites was determined by surveying the ACGME database for the total number of programs listed and the presence or absence of website links. Links, if they were provided, were characterized as either functional or nonfunctional. Functional links led to a website. Nonfunctional links led to an error page. Functional links were then evaluated as being either *direct* (landing directly on the program webpage) or *indirect* (landing on a different page such as the departmental website, requiring further action by the reviewer to access the specific program webpage if possible).

### Availability of Content

Websites for integrated vascular surgery, thoracic surgery, and interventional radiology residency programs were analyzed for availability of information used to inform and recruit prospective applicants. Content items on recruitment and education (listed in Textbox 1) were selected based on ACGME program requirements as well as previously published literature reviewing the online content of residency and fellowship programs [5,14,19,20]. Content on the training program websites was counted as present if it was present on the main training program webpage or it was accessible via a direct link provided on the main training program webpage.

**Content features included in evaluation of integrated vascular surgery, integrated thoracic surgery, and integrated interventional radiology training program websites.**

**Program recruitment (n=41)**

- Program description
- Number incoming positions available
- Faculty listing
- Faculty education and training history
- Faculty profile (descriptive)
- Faculty publications
- Faculty contact information
- Current residents
- Resident education history
- Resident profiles
- Resident contact information
- Alumni listing
- Alumni education history
- Alumni contact information
- Alumni career placement
- Board examination performance
- Program chair message
- Program director message
- Program director contact
- Administrative/coordinator contact
- Facility description
- Application requirements
- Selection process
- Interview dates
- Interview day details
- Electronic Residency Application Service (ERAS) link
- If present, is ERAS link functional?
- Call requirement
- Contract
- Salary
- Work hours
- Benefits
- Vacation
- City information
- Domestic considerations
- Well-being strategies
- Debt management
- Meal allowance
- Educational fund
- Parking
- Visa

**Program education (n=16)**

- Rotation schedule
- Didactic instruction
- Research requirements
- Research interests (department/faculty)
- Operative experience
- Journal club
- Conference schedule
- National/regional meetings attended
- Evaluation criteria
- Faculty mentorship
- National organization link
- Curriculum
- Company link
- Elective rotation
- Simulation training
- Vascular lab

### Program Recruitment and Education

Websites were evaluated for content relevant to program recruitment and education. Program recruitment information included faculty listings, faculty and departmental research interests, alumni career placements, and information on current residents. Recruitment information regarding the application and interview process as well as general resident quality of life metrics were also evaluated (see Textbox 1). Program education content addressed operative and didactic training. It also covered resident research opportunities. Overall, 41 program recruitment and 16 program education content items were evaluated.

### Rater Training and Consistency

Each website was accessed and evaluated by two reviewers (one medical student and one resident) for availability of content items as well as quality of websites (determined as a function of four dimensions: content, design, organization, and user friendliness). Each reviewer was trained by examining an optimal website, an average website, and a below average website with the senior author. Disputed assessments were resolved by consensus following discussion with the senior author. Reviewers were not blinded.

Overall, there was considerable interrater reliability with 81% agreement (*κ*=0.74).

### Data Analysis

Intergroup analysis of continuous variables was performed using ANOVA. Categorical variables were compared using chi-square analysis. Statistical significance was defined as *P*<.05. Percent agreement and kappa statistics were calculated for interrater reliability. Statistical analysis was performed using statistical software R version 4.0.0 (R Foundation for Statistical Computing).

### Ethical Approval

All data reviewed was open to the public, and there was no contact with fellowship staff; thus, no institutional review board review, ethics approval, or informed consent was necessary.

## Results

### Accessibility of Websites

Of the programs included in this analysis, 87% (53/61) of the vascular surgery, 89% (24/27) of the thoracic surgery, and 95% (81/85) of the interventional radiology programs provided a link to their program webpage on the ACGME webpage (*P*=.18). Of those programs that provided links, the majority of the links were functional with no difference between the specialties (*P*=.24). However, few links landed directly on the program webpage. Less than one-third of the programs with functional links provided links that landed directly on the program webpage (*P*=.52). Overall, 56 of 61 (92%) vascular surgery, 27 of 27 (100%) thoracic surgery, and 74 of 85 (87%) interventional radiology programs had a dedicated webpage (Table 1).

**Table 1:** Accessibility of integrated vascular surgery, integrated thoracic surgery, and integrated interventional radiology training program websites from the Accreditation Council for Graduate Medical Education webpage.

	Vascular surgery	Thoracic surgery	Interventional radiology	*P* value
Programs	61	27	85	N/A^a^
Providing website links^b^, n (%)	53 (87)	24 (89)	81 (95)	.18
Functioning links, n (%)	47 (89)	21 (88)	74 (91)	.24
Direct links, n (%)	17 (32)	7 (33)	17 (23)	.52

^a^N/A: not applicable.

^b^Accreditation Council for Graduate Medical Education links were accessed November 2019.

### Availability of Content

Content was assessed in two domains: recruitment and education. Of the 57 recruitment and educational content items included in this analysis, vascular surgery program webpages contained a median of 26 (IQR 20-32) content items, thoracic surgery program webpages contained a median of 27 (IQR 21-32) content items, and interventional radiology program webpages contained a median of 23 (IQR 18-27) content items. Of the 41 recruitment content items included in this analysis, vascular surgery program webpages contained a median of 19.5 (IQR 15-24) content items, thoracic surgery program webpages contained a median of 20 (IQR 16-24) content items, and interventional radiology program webpages contained a median of 18 (IQR 15-21) content items. Of the 16 education content items included in this analysis, vascular surgery program webpages contained a median of 7 (IQR 4-9) content items, thoracic surgery program webpages contained a median of 6 (IQR 4-7) content items, and interventional radiology program webpages contained a median of 4 (IQR 3-7) content items (Table 2).

**Table 2:** Availability of content on US integrated vascular surgery, integrated thoracic surgery, and integrated interventional radiology training program websites.

		Vascular surgery (n=56), n (%)	Thoracic surgery (n=27), n (%)	Interventional radiology (n=74), n (%)	*P* value
**Program recruitment**					
	Program description	56 (100)	26 (96)	69 (93)	.14
	Faculty listing	53 (95)	27 (100)	73 (99)	.23
	Faculty education (training history)	52 (93)	25 (93)	68 (92)	.98
	Admin/coordinator contact	53 (95)	22 (82)	63 (85)	.14
	Faculty profile (descriptive)	46 (82)	19 (70)	68 (92)	*.02* ^a^
	Application requirements	42 (75)	22 (82)	50 (68)	.34
	ERAS^b^ link	35 (63)	16 (59)	47 (64)	.93
	If present, is ERAS link functional?	35 (63)	16 (59)	47 (64)	.93
	Benefits	39 (70)	19 (70)	51 (69)	.99
	Facility description	50 (89)	22 (82)	49 (66)	*.007*
	Number of incoming positions available	44 (79)	20 (74)	45 (61)	.08
	Salary	34 (61)	17 (63)	48 (65)	.89
	Current residents	40 (71)	20 (74)	47 (64)	.49
	Vacation policy	32 (57)	18 (67)	46 (62)	.69
	Program director contact	24 (43)	8 (30)	43 (58)	*.03*
	Faculty publications	24 (43)	19 (70)	40 (54)	.06
	Well-being strategies	26 (46)	17 (63)	37 (50)	.36
	City information	33 (59)	16 (59)	35 (47)	.34
	Educational fund	19 (34)	12 (44)	35 (47)	.30
	Resident education history	35 (63)	17 (63)	34 (46)	.11
	Parking	17 (30)	9 (33)	34 (46)	.16
	Domestic considerations	32 (57)	16 (59)	32 (43)	.19
	Visa	22 (39)	12 (44)	32 (43)	.87
	Interview dates	32 (57)	13 (48)	29 (39)	.13
	Faculty contact information	17 (30)	9 (33)	27 (37)	.76
	Meal allowance	16 (29)	11 (41)	27 (37)	.48
	Sample contract	9 (16)	7 (26)	21 (28)	.25
	Call requirement	22 (39)	16 (59)	21 (28)	*.02*
	Program director message	17 (30)	3 (11)	18 (24)	.16
	Resident profiles	10 (18)	7 (26)	18 (24)	.60
	Interview details	10 (18)	5 (19)	18 (24)	.63
	Alumni listing	20 (36)	7 (26)	17 (23)	.27
	Alumni career placement	15 (27)	6 (22)	17 (23)	.85
	Selection process	4 (7)	2 (7)	13 (18)	.14
	Debt management	22 (39)	11 (41)	8 (11)	*<.001*
	Resident contact information	5 (9)	3 (11)	6 (8)	.90
	Work hours	12 (21)	12 (44)	6 (8)	*<.001*
	Alumni education history	7 (13)	1 (4)	2 (3)	.06
	Alumni contact information	1 (2)	0 (0)	1 (1)	.79
	Program chair message	5 (9)	2 (7)	1 (1)	.13
	Board examination performance	4 (7)	0 (0)	1 (1)	.10
**Program education**					
	Rotation schedule	47 (84)	19 (70)	45 (61)	*.02*
	Research interests (department)	22 (39)	16 (59)	49 (66)	*.008*
	Didactic instruction	39 (70)	17 (63)	42 (57)	.32
	Research requirements	41 (73)	19 (70)	39 (53)	*.04*
	Operative experience	18 (32)	5 (19)	37 (50)	*.008*
	Journal club	34 (61)	11 (41)	25 (34)	*.008*
	Meetings attended	26 (46)	11 (41)	23 (31)	.20
	Elective rotation	25 (45)	12 (44)	20 (27)	.07
	Conference schedule	21 (38)	6 (22)	18 (24)	.19
	Curriculum	21 (38)	10 (37)	12 (16)	*.01*
	Faculty mentorship	6 (11)	3 (11)	11 (15)	.75
	Vascular lab	36 (64)	2 (7)	10 (14)	*<.001*
	National organization link	4 (7)	7 (26)	8 (11)	*.04*
	Evaluation criteria	9 (16)	6 (22)	5 (7)	.08
	Simulation training	19 (34)	9 (33)	3 (4)	*<.001*
	Company link	2 (4)	1 (4)	0 (0)	.26

^a^Italics indicate significant values.

^b^ERAS: Electronic Residency Application Service.

### Vascular Surgery

For program recruitment, almost all programs provided information on program description, faculty listing, faculty education, administrator or coordinator contact information, facility description, descriptive faculty profiles, and the number of incoming positions. The majority of programs provided information on application requirements, a functional link to the Electronic Residency Application Service (ERAS), benefits, salary, current residents, city information, resident education history, domestic considerations, vacation policy, and interview dates. Less than one-half of programs provided information on program director contact information, faculty publications, well-being strategies, faculty contact information, program director message, alumni listing, alumni career placement, educational fund, parking, nonnational visa information, meal allowance, call requirement, alumni career placement, and debt management. Fewer than one-quarter of the programs provided information on sample contracts, resident profiles, interview details, work hours, and alumni education history. Almost no programs provided information on their selection process, resident contact information, program director message, board examination performance, and alumni contact information (Table 2).

For program education, almost all programs provided information on rotation schedule (84%; 47/56). The majority of programs provided information on didactic instruction, research requirements, journal club, and vascular lab training (Registered Physician in Vascular Interpretation [RPVI]). Less than one-half of programs provided information on departmental research interests, operative experience, meetings attended, elective rotations, conference schedule, curriculum, and simulation training. Fewer than one-quarter of the programs provided information on evaluation criteria and faculty mentorship. Almost no programs provided information on national organizational links and cardiovascular product company links (Table 2).

### Thoracic Surgery

For program recruitment, almost all programs provided information on program description, faculty listing, faculty education, administrator or coordinator contact information, facility description, and application requirements. The majority of programs provided information on descriptive faculty profiles, a functional link to ERAS, benefits, the number of incoming positions available, salary, current residents, faculty publications, well-being strategy, city information, resident education history, vacation policy, call requirements, and domestic considerations. Less than one-half of programs provided information on program director contact information, interview date, faculty contact information, sample contracts, resident profiles, alumni listings, debt management, educational fund, parking, nonnational visa information, meal allowance, and work hours. Fewer than one-quarter of the programs provided information on program director message, alumni career placement, interview details, and resident contact information. Almost no programs provided information on selection process, alumni education history, program chair message, board examination performance, and alumni contact information (Table 2).

For program education, the majority of programs provided information on rotation schedule, departmental research interests, didactic instruction, and research requirements. Less than one-half of programs provided information on journal club, meetings attended, elective rotation, curriculum, national organization links, and simulation training. Fewer than one-quarter of the programs provided information on operative experiences, conference schedule, faculty mentorship, and evaluation criteria (Table 2). Almost no programs provided information on vascular lab training (RPVI), which should be expected as it is not a core component of thoracic surgery.

### Interventional Radiology

For program recruitment, almost all programs provided information on program description, faculty listing, faculty education, administrator or coordinator contact information, and descriptive faculty profiles. The majority of programs provided information on application requirements, a functional link to ERAS, facility description, vacation policy, benefits, the number of incoming positions available, salary, current residents, program director contact information, and faculty publications. Less than one-half of programs provided information on well-being strategies, city information, educational fund, parking, nonnational visa information, meal allowance, call requirement, resident education history, domestic considerations, interview dates, faculty contact information, and sample contracts. -quarter of the programs provided information on a program director message, resident profiles, interview details, alumni listing, alumni career placement, selection process, and debt management. Almost no programs provided information on resident contact information, work hours, alumni education history, program chair message, board examination performance, and alumni contact information (Table 2).

For program education, the majority of programs provided information on rotation schedule, departmental research interests (49/74, 66%), didactic instruction, and research requirements. Less than one-half of programs provided information on operative experiences, journal club, meetings attended, and elective rotations. Fewer than one-quarter of the programs provided information on conference schedule, curriculum, faculty mentorship, vascular lab training (RPVI), and national organization links. Almost no programs provided information on evaluation criteria, simulation training, and cardiovascular product company links (Table 2).

### Comparison of Content Availability

Vascular surgery webpages provided the most information on rotation schedule, journal club, and vascular lab (as compared to thoracic surgery and interventional radiology webpages (*P*=.02, *P*=.008, and *P*<.001, respectively). Vascular surgery webpages provided less information on departmental research interests as compared to thoracic surgery and interventional radiology webpages (*P*=.008; Table 2).

Thoracic surgery webpages provided the most information on call requirement, national organization link, and work hours as compared to vascular surgery and interventional radiology webpages (*P*=.02, *P*=.04, and *P*<.001, respectively). Thoracic surgery webpages provided less information on descriptive faculty profile as compared to vascular surgery and interventional radiology webpages (*P*=.02; Table 2).

Interventional radiology webpages provided the most information on operative experience and program director contact information as compared to vascular and thoracic surgery webpages (*P*=.008 and *P*=.03, respectively). Interventional radiology webpages provided less information on facility description, debt management (*P*=.007), research requirements (*P*<.001), curriculum (*P*=.04), and simulation training (*P*<.001) as compared to vascular surgery and thoracic surgery webpages (Table 2).

### Quality of Websites

On an overall assessment, integrated vascular surgery, thoracic surgery, and interventional radiology websites were found to be comparable. The average vascular surgery website score was 2.66 (SD 0.95), the average thoracic surgery website score was 2.18 (SD 0.92), and the average interventional radiology website score was 2.25 (SD 0.88). The vascular surgery websites had the highest scores in content, design, organization, and user-friendliness. The thoracic surgery websites had the lowest scores in content, organization, and user-friendliness, while the interventional radiology websites had the lowest score in design. Additional details regarding website quality, broken down by category, are visible in Table 3.

**Table 3:** Quality of US integrated vascular surgery, integrated thoracic surgery, and integrated interventional radiology training program websites^a^.

	Content, mean (SD)	Design, mean (SD)	Organization, mean (SD)	User friendliness, mean (SD)	Average quality, mean (SD)
Vascular surgery	2.57 (0.95)	2.59 (0.99)	2.75 (0.96)	2.73 (0.90)	2.66 (0.95)
Thoracic surgery	2.04 (0.85)	2.22 (0.89)	2.19 (1.08)	2.26 (0.86)	2.18 (0.92)
Interventional radiology	2.27 (0.90)	2.05 (0.77)	2.32 (0.97)	2.34 (0.88)	2.25 (0.88)

^a^Scale: 1=poor, 2=acceptable, 3=good, 4=great.

## Discussion

### Principal Findings

As resident recruitment moves to a virtual platform, the internet is an increasingly important resource for residency applicants as they research programs. Thoracic surgery program webpages had the most information, followed by vascular surgery program webpages, with interventional radiology program webpages reporting the least content. This trend in availability of content items mirrors the percent of positions filled by each specialty, with 100% of PGY-1 thoracic surgery positions filled, 97% of vascular surgery PGY-1 positions filled, and 97% of PGY-1 interventional radiology positions filled (with 94% of PGY-2 interventional radiology positions filled), as reported by the NRMP 2020 Main Residency Match Results and Data report [28].

**Figure 1:**
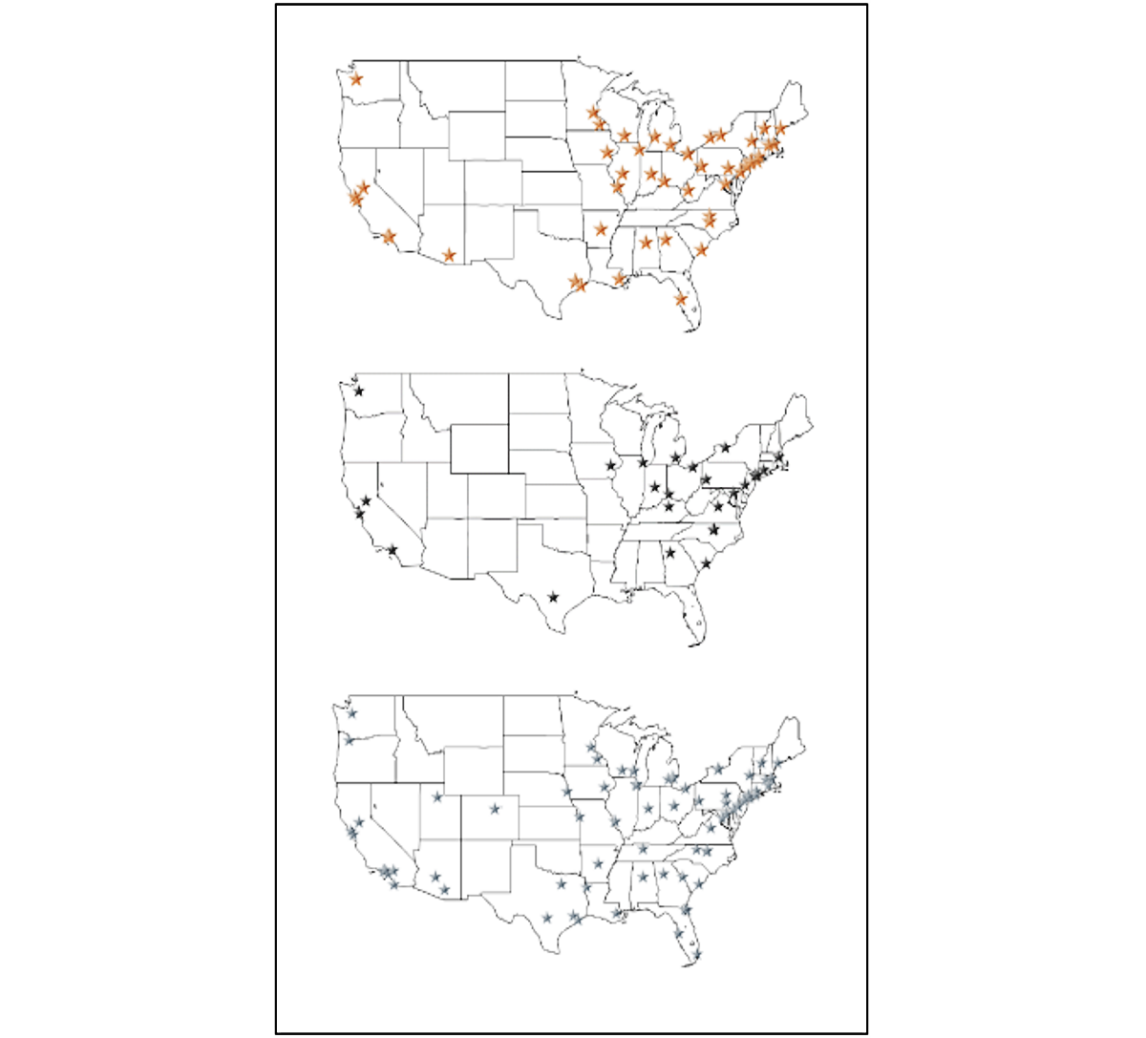
Map of integrated vascular surgery (orange), thoracic surgery (black), and interventional radiology (silver) training programs.

Other factors, beyond program websites, that have been identified to influence applicant interest in a program include geography, advice from a mentor, advice from a peer, and other online information. The integrated vascular track was first accredited by the ACGME in 2006 [27], the first integrated thoracic surgery program accepted residents in 2007 [29], and the first integrated interventional radiology programs participated in the NRMP in 2016 [30]. The majority of these integrated programs have been established for less than 10 years. This increase in the number of integrated programs, though necessary to meet the high demand for integrated residency positions, means that many programs do not have an established national presence. Applicants cannot receive the same quality of advice from mentors and peers on newer programs, as compared to programs that have been established for longer periods of time. Furthermore, many programs are geographically clustered, specifically in the northeast and along the west coast (see Figure 1). These factors combine to place additional weight on program websites, perhaps serving as the initial source of information for potential applicants and allowing for comparison.

Of ACGME-accredited programs, 56 of 61 (92%) vascular surgery programs, 27 of 27 (100%) thoracic surgery programs, and 74 of 85 (87%) interventional radiology programs had functional websites. Thoracic surgery program webpages had the most information (content item median 27, IQR 21-32), then vascular surgery program webpages (content item median 26, IQR 20-32), with interventional radiology program webpages reporting the least content (content item median 23, IQR 18-27). The greater amount of content on vascular surgery and thoracic surgery program webpages could be expected, given the young age of many interventional radiology programs. Previous studies have acknowledged integrated interventional radiology program webpages to be a work in progress [15]. This study confirms that finding in relation to longer-established vascular surgery and thoracic surgery program webpages.

Two content items that have been identified to be highly influential to the applicant program decision are operative experience and faculty mentorship [31-33]. This analysis found those items to be reported at 32% (18/56) and 11% (6/56) for vascular surgery, 19% (5/27) and 11% (3/27) for thoracic surgery, and 50% (37/74) and 15% (11/74) for interventional radiology programs (*P*=.008 and *P*=.75), respectively. Additional notable deficits for vascular surgery websites were resident profiles, sample contracts, and departmental research interests. Thoracic surgery websites lacked program director contact information and message as well as information on operative experience. Interventional radiology websites had deficits in work hours, debt management, and curriculum. All specialty websites had deficits in evaluation criteria and faculty mentorship. In addition to addressing the deficits in program recruitment and education content items, the deficits in lifestyle management cannot be disregarded; medical students increasingly report controllable lifestyle as a major factor in specialty choice [34,35].

The deficits identified by this analysis are comparable to deficits identified for other specialties. Other studies have found considerable deficits in newsletter, resident listings and photographs, faculty contact information, and away elective rotation information for dermatology websites [3]; resident call schedule, alumni career placement, and salary for orthopedic surgery websites [7]; academic conference schedule, call schedule, operative case listing, graduate fellowship information, and board exam performance for plastic surgery websites [19]; evaluation criteria, call schedule, operative exposure, national meetings attended, debt management, alumni contact, and work hours for neurosurgery websites [20]; call schedule, away elective rotation information, resident profiles, and faculty research for general surgery websites [21]; and call schedule, active/past research projects, area information, message from the program director or chair, selection criteria, salary, and surgical statistics for otolaryngology websites [22].

Overall, we recommend that programs address the deficits in specific content items identified by this analysis. Given the increasingly important role of online information in the residency application process and the anticipated transition to a virtual application process for the 2021 cycle, it would behoove programs to increase their online presence. In addition to the content items included in this analysis, it might be fitting for programs to include more personal information (ie, more detailed resident and attending profiles) to give applicants a better idea of the personality of different programs, replacing the role previously served by in-person away rotations and interviews.

This study had several limitations. First, this data is representative of what information was available online at the time of data collection. It is possible that websites could have been edited or new program websites could have been published since that time. Additionally, though an extensive list of content items were evaluated by reviewers regarding program recruitment and education, it is possible that other unmentioned content items could hold bearing on an applicant’s decision. Finally, reviewers were not blinded to what program they were evaluating. Thus, any inherent bias reviewers might have had for particular programs was not controlled for. The nature of this study did not lend itself to evaluating the association between website content, to what specialty and to what programs applicants apply, and ultimate applicant program placement. Future studies could seek to characterize this trajectory.

### Conclusion

This study has uncovered key differences in the availability of online content for residency programs recruiting from the same pool of applicants. Thoracic surgery program webpages have the most information, then vascular surgery program webpages, with interventional radiology program webpages reporting the least content. Recruitment is highly competitive between vascular surgery, thoracic surgery, and interventional radiology with the ratio of applicants to positions being highest for thoracic surgery, then interventional radiology, and lastly vascular surgery, as reported by ERAS. To attract valuable candidates, programs should aim to increase the availability of online content for potential applicants.

## Abbreviations

ACGME: Accreditation Council for Graduate Medical Education
ERAS: Electronic Residency Application Service
NRMP: National Resident Matching Program
RPVI: Registered Physician in Vascular Interpretation
